# DDX56 Binds to Chikungunya Virus RNA To Control Infection

**DOI:** 10.1128/mBio.02623-20

**Published:** 2020-10-27

**Authors:** Frances Taschuk, Iulia Tapescu, Ryan H. Moy, Sara Cherry

**Affiliations:** a Department of Pathology and Laboratory Medicine, University of Pennsylvania, Philadelphia, Pennsylvania, USA; b Cell and Molecular Biology Graduate Group, University of Pennsylvania, Philadelphia, Pennsylvania, USA; c Biochemistry and Biophysics Graduate Group, University of Pennsylvania, Philadelphia, Pennsylvania, USA; d Immunology Graduate Group, University of Pennsylvania, Philadelphia, Pennsylvania, USA; University of Colorado School of Medicine

**Keywords:** alphavirus, chikungunya, Sindbis, CLIP-Seq, DEAD-box helicase, RNAi, CLIP-seq, DEAD-box, RNA binding, RNA virus, antiviral, arbovirus, genomic RNA, helicase

## Abstract

DEAD box RNA helicases regulate diverse facets of RNA biology. Proteins of this family carry out essential cellular functions, and emerging literature is revealing additional roles in immune defense. Using RNA interference screening, we identified an evolutionarily conserved antiviral role for the helicase DDX56 against the alphavirus Sindbis virus (SINV), a mosquito-transmitted pathogen that infects humans. Depletion of DDX56 enhanced infection in *Drosophila* and human cells. Furthermore, we found that DDX56 also controls the emerging alphavirus chikungunya virus (CHIKV) through an interferon-independent mechanism. Using cross-linking immunoprecipitation (CLIP-Seq), we identified a predicted stem-loop on the viral genomic RNA bound by DDX56. Mechanistically, we found that DDX56 levels increase in the cytoplasm during CHIKV infection. In the cytoplasm, DDX56 impacts the earliest step in the viral replication cycle by binding and destabilizing the incoming viral genomic RNA, thereby attenuating infection. Thus, DDX56 is a conserved antiviral RNA binding protein that controls alphavirus infection.

## Abstract (precis)

Arthropod-borne viruses are diverse pathogens and include the emerging virus chikungunya virus, which is associated with human disease. Through genetic screening, we found that the conserved RNA binding protein DDX56 is antiviral against chikungunya virus in insects and humans. DDX56 relocalizes from the nucleus to the cytoplasm, where it binds to a stem-loop in the viral genome and destabilizes incoming genomes. Thus, DDX56 is an evolutionarily conserved antiviral factor that controls alphavirus infection.

## INTRODUCTION

Innate immune defenses rely on the recognition of invading pathogens by the host cell. In the case of RNA viruses, recognition is largely driven by RNA binding proteins that recognize foreign structures on viral RNAs (1). Key sensors of viral RNA, including the canonical cytosolic RNA sensors, retinoic acid-inducible gene I (RIG-I/DDX58) and melanoma differentiation-associated protein 5 (MDA-5/IFIH1), are members of the DExD/H family of RNA binding proteins (2). These proteins are closely related to the larger DEAD box helicase family, which is the largest RNA helicase family and is deeply conserved. DEAD box helicases share a series of characteristic motifs, comprising the helicase core domain, but have diverse functions representing every stage of cellular RNA metabolism (3), although the precise cellular roles of many remain poorly defined.

DEAD box helicases recognize the phosphate backbone of RNA rather than the nucleotide bases, suggesting that they bind RNAs with a sequence-independent but structure-dependent mechanism (4). Although paralogous members of the DEAD box helicase family have diverse N- and C-terminal regions, orthologous proteins tend to be highly conserved (5). DEAD box helicase binding to target nucleic acids can have diverse outcomes: acting as a platform to recruit additional proteins, mediating RNA unwinding, and/or inducing protein-RNA remodeling. Many of these functions are dependent on ATPase- and ATP-dependent helicase activities (3).

In addition to the canonical cytoplasmic RNA sensors RIG-I and MDA5, emerging data suggest that many DEAD box helicases also have roles in viral RNA recognition and cellular antiviral responses. DDX1, DDX21, DHX36, and the adapter protein TRIF form a complex in the cytosol, which binds double-stranded RNA (dsRNA), contributing to interferon (IFN) induction (6). Other helicases, including DDX60, DDX3, and DHX9, also contribute to RNA sensing and interferon induction (7–9). DDX41 is involved in the sensing of dsDNA and activates cellular responses to diverse intracellular pathogens, including bacteria and DNA viruses (10). DEAD box helicases can also control infection independently of interferon signaling; DDX17 binds an essential stem-loop structure on Rift Valley fever virus RNA to control infection (11). It is likely that many other helicases have uncharacterized antiviral activity.

Alphaviruses are a large group of enveloped single-stranded, positive-sense RNA viruses of about 11 to 12 kb. Alphaviruses that infect humans are transmitted by vector mosquitoes and include Sindbis virus (SINV) and chikungunya virus (CHIKV). SINV is the prototypical alphavirus, and it typically causes mild infection in humans. After initial outbreaks in Africa, SINV spread to Europe in the 1960s and now has seroprevalence of around 5% in Finland (12). CHIKV was first identified during an outbreak in present-day Tanzania in 1952 (13, 14) and has been reemerging since 2004, spreading around the globe (15). CHIKV infection is usually symptomatic, causing fever, rash, and severe joint pain, which can persist even after infection has cleared (15). Alphaviruses such as CHIKV pose a threat to human health, and no vaccines or specific antivirals are available to alleviate their disease burden.

Different alphaviruses replicate using the same general mechanisms (16). Virions enter cells by clathrin-mediated endocytosis, and fusion in the endosome deposits viral RNA into the cytoplasm. This incoming genomic RNA is capped and polyadenylated, and to launch infection, it must be directly translated by cellular machinery upon uncoating. This first round of translation produces the nonstructural polyprotein nsP1234. The early steps of translation are facilitated by structural features in the RNA, including a conserved stem-loop (17). Sequential cleavage of the nsP1234 polyprotein by the protease activity of nsP2 activates the resulting complex for RNA replication, leading first to the synthesis of the negative-stranded antigenomic RNA and then the positive-sense genomic RNA. As the replication complex is processed further, a second, subgenomic positive-sense RNA transcript is produced from the antigenome. This subgenomic RNA is produced in ∼10-fold excess of the genome and is translated into the structural polyprotein, which is processed into the structural proteins required for the assembly and egress of progeny viral particles. While alphaviruses are known to be sensed by RIG-I and sensitive to type I interferons, these viruses also encode antagonists that block these pathways (18–20). Therefore, it is likely that interferon-independent mechanisms also play an important role in the control of these infections.

We set out to determine if there were previously uncharacterized antiviral functions for DEAD box helicases in alphavirus infection. Because alphaviruses are transmitted by insect vectors, we reasoned that there may be antiviral helicases conserved in both insects and humans that impact infection. Moreover, because insects lack an interferon system, identifying conserved antiviral proteins could reveal interferon-independent mechanisms of viral control. To this end, we performed an RNA interference (RNAi) screen of conserved DEAD box helicases in *Drosophila* cell culture and found that Hlc (dDDX56) is antiviral against SINV. DDX56 is a DEAD box helicase with known nucleolar localization and a role in ribosome biogenesis (21), but a role in antiviral immunity has not been previously described. We tested whether this antiviral function was conserved in human DDX56 and found that this protein is antiviral against SINV and CHIKV in human cells.

We found that DDX56 is dispensable for interferon induction, leading us to hypothesize that DDX56 binds viral RNA to control infection more directly. We used cross-linking immunoprecipitation (CLIP-Seq) to immunoprecipitate RNAs bound by DDX56 during CHIKV infection of human cells. Strikingly, we found that approximately 10% of the DDX56-bound RNAs were viral sequences. Moreover, we observed a strong enrichment of DDX56 binding within the coding region of the CHIKV RNA-dependent RNA polymerase (RdRp; nsP4). This peak is adjacent to a highly conserved hairpin motif that promotes the translation of the RdRp, a step required to launch productive infection (17, 22). This led us to hypothesize that DDX56 binding to this region would impact very early steps in the replication cycle, perhaps prior to translation of the RdRp. Indeed, we found that DDX56 controls infection at the earliest stages, impacting the stability of incoming genomic RNA. Altogether, we have defined a new role for the DEAD box helicase DDX56 in the control of alphavirus infection, which is conserved from insects to humans.

## RESULTS

### DDX56 is antiviral.

We hypothesized that DEAD box helicases have previously unknown antiviral activities against alphaviruses. We used double-stranded RNA (dsRNA) to knock down a panel of conserved DEAD box helicases (11) in *Drosophila* DL1 cells and infected these cells with a green fluorescent protein (GFP) reporter strain of Sindbis virus (SINV-GFP); 3 days postinfection, levels were quantified using microscopy with automated image analysis. We used dsRNA against the GFP reporter as a positive control for knockdown and a nontargeting dsRNA as a negative control. We identified antiviral helicases using a cutoff of >2-fold increase in infection; of the 22 DEAD box helicases in our panel, six exceeded this threshold, and five of those had no impact on cell survival (see Fig. S1A to C in the supplemental material).

10.1128/mBio.02623-20.1 FIG S1 DDX56 is antiviral. (A and B) A panel of *Drosophila* RNA helicase genes was depleted by RNAi in DL1 cells. (A) Cells were infected with SINV-GFP at day 3 and fixed at 24 h postinfection. Percent infection was quantified by automated microscopy and expressed as fold change in percent infection compared to a nontargeting control. Graph shows mean and SEM from three replicates. (B) Quantification of cell numbers from panel A, quantified (number of nuclei) relative to a nontargeting control. Mean and SEM from three replicates are shown. (C) Representative microscopy images from panel A. Signal from SINV-GFP reporter virus is displayed in green, and Hoechst nuclear counterstain is in blue. (D) U2OS cells were transfected with pooled or individual siRNAs to DDX56. Infection with CHIKV was assessed by automated microscopy. Graph shows mean and SEM; *, *P* < 0.05. (E) U2OS cells were transfected with “control, IFIT1, or DDX56” siRNAs, and infection with CHIKV was assessed by RT-qPCR normalized to GAPDH and by automated microscope. Graphs show mean and SEM. *, *P* < 0.05; **, *P* < 0.01. Download FIG S1, PDF file, 0.1 MB. Copyright © 2020 Taschuk et al. 2020 Taschuk et al. This content is distributed under the terms of the Creative Commons Attribution 4.0 International license.

**FIG 1:**
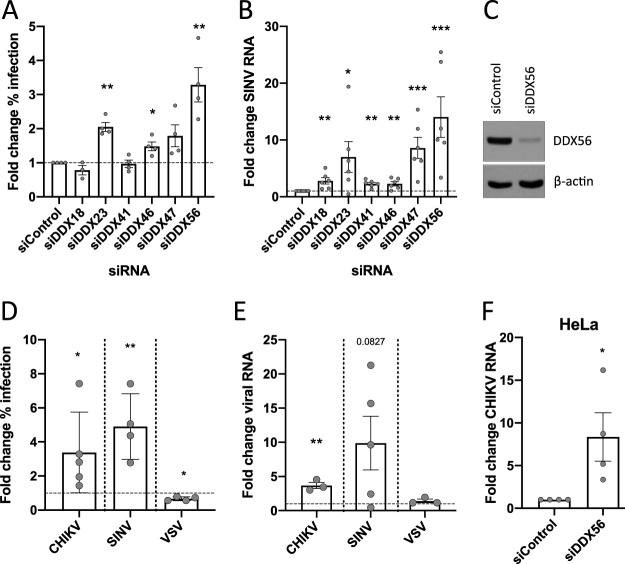
DDX56 is antiviral. (A and B) A panel of conserved DEAD box helicases was targeted by RNAi in human cells. U2OS cells were transfected with the indicated siRNAs and infected 3 days later with Sindbis virus expressing GFP (SINV-GFP). (A) For analysis of percent infection, the fraction of cells infected was quantified by automated scoring of microscopy images and is expressed as fold difference compared to cells treated with a nontargeting control siRNA. Data are from 4 independent sets of siRNA knockdown and infection; graphs show means and standard errors of the means (SEM). (B) Relative viral RNA was quantified by RT-qPCR normalized to 18s rRNA and compared to cells treated with a nontargeting control. Graphs show means and SEM from 6 replicate transfection experiments. (C) U2OS cells were transfected with control or DDX56 siRNAs and subjected to immunoblot analysis with β-actin as a loading control. Representative is blot shown, *n* = 3. (D and E) U2OS cells were transfected with control or DDX56 siRNAs and infected with Sindbis virus (SINV), chikungunya virus (CHIKV), or vesicular stomatitis virus (VSV). Infection was quantified by automated microscopy (D) or RT-qPCR normalized to GAPDH (E). Data are expressed as fold change in DDX56-depleted cells compared to cells treated with a nontargeting control, and dots represent individual knockdown experiments. Graphs show means and SEM. (F) HeLa cells were transfected with control or DDX56 siRNAs and infected with CHIKV. Differences in infection were quantified by RT-qPCR for 4 independent knockdown experiments. Graphs show means and SEM. *, *P* < 0.05; **, *P* < 0.01. See also Fig. S1.

We next tested a panel of human orthologs of the antiviral *Drosophila* helicases, using the human osteosarcoma cell line U2OS, as it is readily infected by many arthropod-borne viruses (arboviruses) and has an intact interferon system. We used short interfering RNA (siRNA) transfection to knock down DEAD box helicase genes and evaluated the effect on SINV-GFP infection by reverse transcription-quantitative PCR (RT-qPCR) and microscopy (Fig. 1A and B). We observed that DDX23, DDX46, and DDX56 were antiviral in both assays, and we observed the strongest antiviral phenotype for DDX56.

To evaluate the breadth of DDX56’s antiviral effect, we challenged control or DDX56-depleted U2OS cells with SINV or the related alphavirus chikungunya virus (CHIKV) as well as the unrelated negative-sense RNA virus vesicular stomatitis virus (VSV). We found that DDX56 was antiviral against both alphaviruses but not VSV using both automated microscopy and RT-qPCR to quantify viral infection in U2OS cells (Fig. 1D and E) and was also antiviral against CHIKV in HeLa cells (Fig. 1F). We verified efficient depletion of endogenous DDX56 by immunoblotting (Fig. 1C) and validated that the siRNAs were on-target by testing them independently; each was individually active against CHIKV infection (Fig. S1D). The increase in CHIKV infection following knockdown of DDX56 was comparable to the effect of knocking down the anti-alphaviral factor IFIT1 (Fig. S1E).

### DDX56 is not required for interferon signaling or ISG expression.

**FIG 2:**
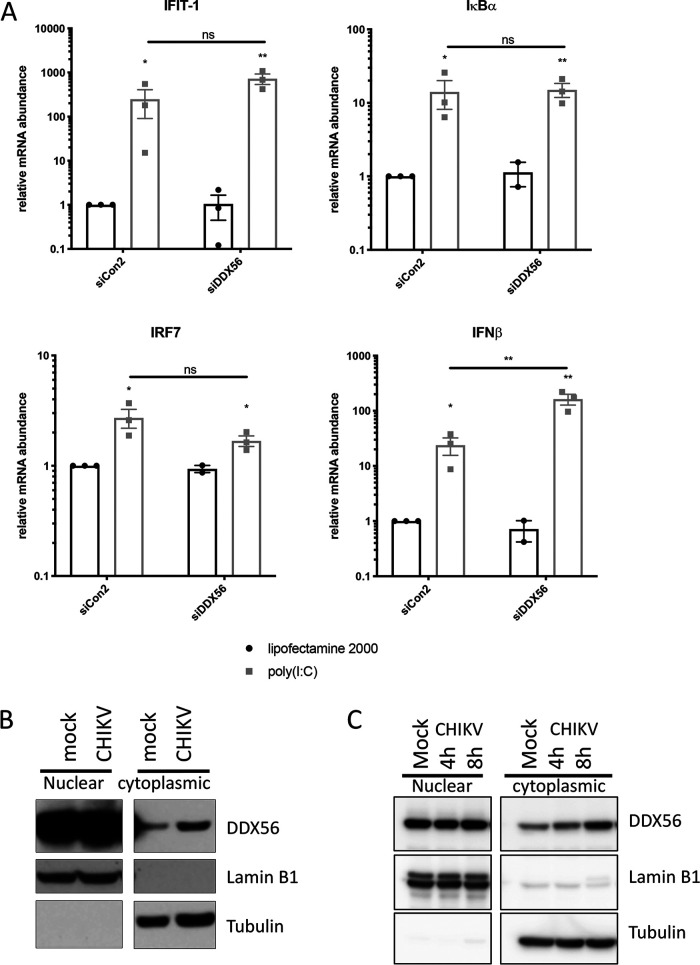
(A) U2OS cells were transfected with control or DDX56 siRNAs. At day 3, cells were transfected with 2.5 μg/ml poly(I·C) or vehicle control. Innate immune gene expression at 8 h after poly(I·C) transfection was quantified by RT-qPCR. Data are normalized to GAPDH and expressed as fold change compared to vehicle-treated cells with nontargeting control siRNA. Statistical significance was assessed by one-sample *t* test on log-transformed fold change. *N* = 3 for all except siDDX56 vehicle control (*N* = 2). (B and C) CHIKV-infected U2OS cells were subjected to nuclear-cytoplasmic fractionation at 24 hpi (B) or at the indicated time points (C). Samples were immunoblotted and probed for DDX56 with lamin B1 and tubulin as controls for fractionation purity. Representative blots are shown, *n* = 3.

The role of type I interferons and interferon-stimulated genes (ISGs) in controlling alphavirus infection is well established (20, 23, 24). Therefore, we investigated whether DDX56 contributes to interferon expression or ISG induction in human cells. We transfected cells with the dsRNA mimic poly(I·C) to activate cytoplasmic RNA sensing pathways in the absence of infection. We examined the effect of DDX56 depletion on expression of IFN-β and the ISGs IFIT1 and IRF7, as well as the NF-κB target IκBα, using RT-qPCR. We found that each of these genes is induced by poly(I·C), but DDX56 is not required for induction; this suggests that the antiviral effect of DDX56 is independent of canonical innate immune pathways (Fig. 2A).

### DDX56 localizes in the cytoplasm during infection.

DDX56 is a nucleolar protein that has been implicated in ribosome biogenesis (21). Viral infections can lead to the relocalization of cellular factors either as a cellular response to infection or due to viral manipulation of cellular processes. To determine whether the localization of DDX56 changes during infection, we fractionated uninfected or CHIKV-infected cells at 20 h postinfection (hpi) and assessed levels of DDX56 in nuclear and cytoplasmic compartments by immunoblotting, using the nuclear protein Lamin B1 and the cytoplasmic protein tubulin as controls for purity. As expected, we observed that the majority of DDX56 is present in the nuclear fraction. However, we found that upon CHIKV infection there is an accumulation of DDX56 in the cytoplasm (Fig. 2B), and we could observe this accumulation as early as 8 hpi (Fig. 2C). These data suggest that viral infection affects the localization of DDX56, leading to accumulation in the cytoplasm during early steps of the infectious cycle.

### DDX56 binds viral RNA.

**FIG 3:**
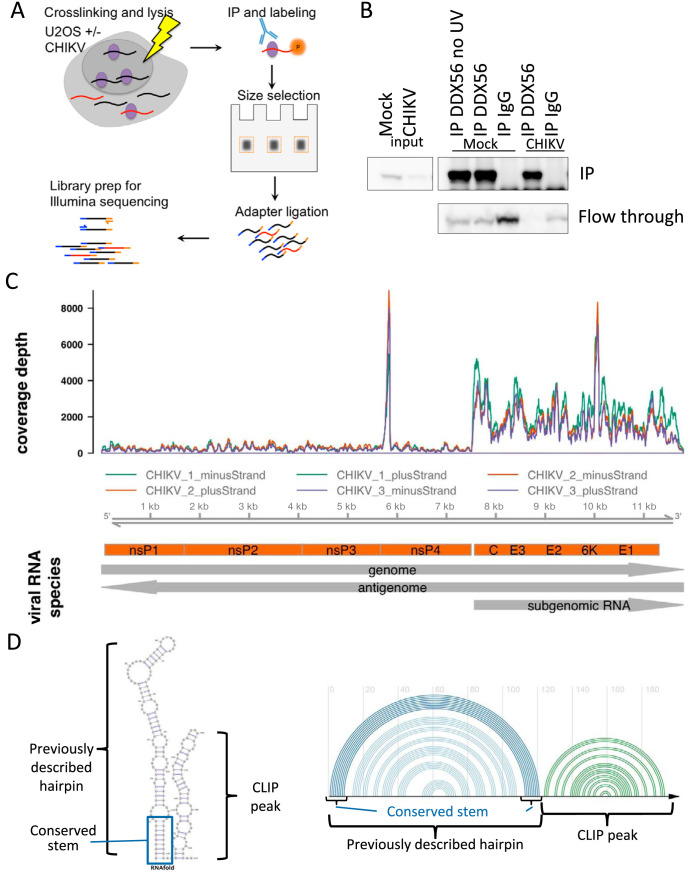
DDX56 is present in the cytoplasm and binds CHIKV RNA. (A) Brief schematic of CLIP-Seq procedure. CHIKV-infected U2OS cells were UV cross-linked at 20 hpi. DDX56 was immunoprecipitated, and copurified RNA was sequenced. (B) Immunoprecipitations of DDX56 show efficient depletion from the flowthrough. (C) Alignment of CLIP-Seq reads on CHIKV genome. Graph shows coverage depth after collapsing PCR duplicates and subtracting signal from negative controls. Three replicates are shown in different colors; reads aligning to the negative strand are graphed as negative numbers. CHIKV RNA species are illustrated below. (D) RNAfold secondary structure prediction of the known CHIKV hairpin and adjacent CLIP peak region expressed as a 2D structure (left) and an arc diagram, where the viral RNA sequence is on the *x* axis and arcs indicate base-pairing interactions (right). See also Fig. S2 and Tables S1 and S2.

DEAD box helicases largely bind structural features on RNA rather than specific sequences, making them suitable as viral sensors because of their potential to interact with conserved structural elements on diverse viruses. Since we observed localization of DDX56 in the cytoplasm, where alphavirus RNA replicates, we set out to determine whether DDX56 binds viral RNA. To identify the RNAs bound by DDX56 during CHIKV infection, we performed CLIP-Seq as previously described (11, 25, 26). Briefly, we infected U2OS cells with CHIKV and treated them with UV light at 20 hpi to cross-link proteins to their bound RNAs. We performed immunoprecipitation using an antibody to endogenous DDX56, purified bound RNAs, and prepared libraries for sequencing on Illumina HiSeq. A schematic is shown in Fig. 3A, and validation of immunoprecipitation with the DDX56 antibody is shown in Fig. 3B. We sequenced libraries from three independent experiments with mock-infected and IgG controls for specificity. We processed resulting sequence files by trimming adapter sequences, collapsing exact duplicates within each sample, and filtering for quality before mapping reads to the human and viral genomes (human, hg19; CHIKV Ross strain, GenBank accession no. AF490259) using STAR (27). Potential cross-contaminating reads on the CHIKV genome were excluded, and PCR duplicates were collapsed using CLIP Tool Kit (28). In CHIKV-infected samples, we found that approximately 10% of the reads were viral sequences, yielding a total of 500,549 viral reads from three replicates. Given that only a small fraction of DDX56 can be found in the cytoplasm, this suggests that much of the cytoplasmic DDX56 pool was bound to viral RNA.

10.1128/mBio.02623-20.2 FIG S2 DDX56 binds a conserved stem loop in CHIKV. (A) Arc diagram of structure prediction from Fig. 2D with conservation analysis. Arc color represents conservation of base-pairing interactions among the strains listed, and colored blocks in the multiple-sequence alignment indicate whether each strain has valid base pairs at those positions. (B) RNAfold prediction of secondary structure for region of SINV genome corresponding to CHIKV in Fig. 2D. Base interactions are represented as an arc diagram and as a 2D folded structure. Download FIG S2, PDF file, 0.1 MB. Copyright © 2020 Taschuk et al. 2020 Taschuk et al. This content is distributed under the terms of the Creative Commons Attribution 4.0 International license.

10.1128/mBio.02623-20.5 TABLE S1 CLIP-Seq coverage depth on CHIKV genome showing positive-strand alignments. Per-base coverage depth of CLIP-Seq reads on CHIKV genome; positive-strand alignments are reported as positive values (CHIKV_coverage_plus.xlsx). Data shown are related to Fig. 3C. Download Table S1, XLS file, 0.3 MB. Copyright © 2020 Taschuk et al. 2020 Taschuk et al. This content is distributed under the terms of the Creative Commons Attribution 4.0 International license.

10.1128/mBio.02623-20.6 TABLE S2 CLIP-Seq coverage depth on CHIKV genome showing negative-strand alignments. Per-base coverage depth of CLIP-Seq reads on CHIKV genome; negative-strand alignments are reported as negative values (CHIKV_coverage_minus.xlsx). Data shown are related to Fig. 3C. Download Table S2, XLS file, 0.3 MB. Copyright © 2020 Taschuk et al. 2020 Taschuk et al. This content is distributed under the terms of the Creative Commons Attribution 4.0 International license.

Analysis of read distribution on the CHIKV genome revealed a sharp peak of coverage near the beginning of the nsP4 gene (approximately positions 5779 to 5852 of AF490259). We also observed greater coverage depth at the 3′ end of the genome than at the 5′ end, corresponding to the subgenomic RNA, and very few reads mapping to the antigenome (a total of 37 reads from 3 replicates). The relative enrichment of 3′-end coverage and lack of antigenome sequences may be related to the relative abundance of these RNA species during infection; very little antigenome is produced relative to genome, while subgenomic RNA is produced in excess (16, 29).

We focused on the sharp peak within the nonstructural protein coding sequence for further exploration. Previous studies defined the region just 5′ of this peak as a highly conserved region, with conserved base pairing across many diverse alphaviruses, including SINV and CHIKV (17). This region is at the 5′ end of nsp4, and they found that mutations in this putative stem impacted nsp4 translation of the alphavirus Venezuelan equine encephalitis virus (17). In CHIKV, SHAPE analysis reveals that the genomic RNA forms a bulged hairpin secondary structure in that region (22), suggesting that this region is essential for viral replication. Our CLIP peak is immediately adjacent to this region; therefore, we used RNAfold (30) to predict the secondary structure of this region as a whole, encompassing both the predicted stem-loop and the CLIP peak. RNAfold prediction identified a clear stem and hairpin in the previously identified conserved structural region, which indicates that the downstream region bound by DDX56 also folds into a structured element, which we have depicted in Fig. 3D as both a two-dimensional (2D) structure and as an arc diagram to indicate the base-pairing interactions (Fig. 3D). Importantly, these base-pairing interactions are conserved in diverse CHIKV strains (Fig. S2A). Moreover, the corresponding region in SINV folds into a similar structure (Fig. S2B). These data suggest that DDX56 binds structural elements of the genomic RNA and that this may regulate infection.

### DDX56 limits CHIKV infection early in infection.

**FIG 4:**
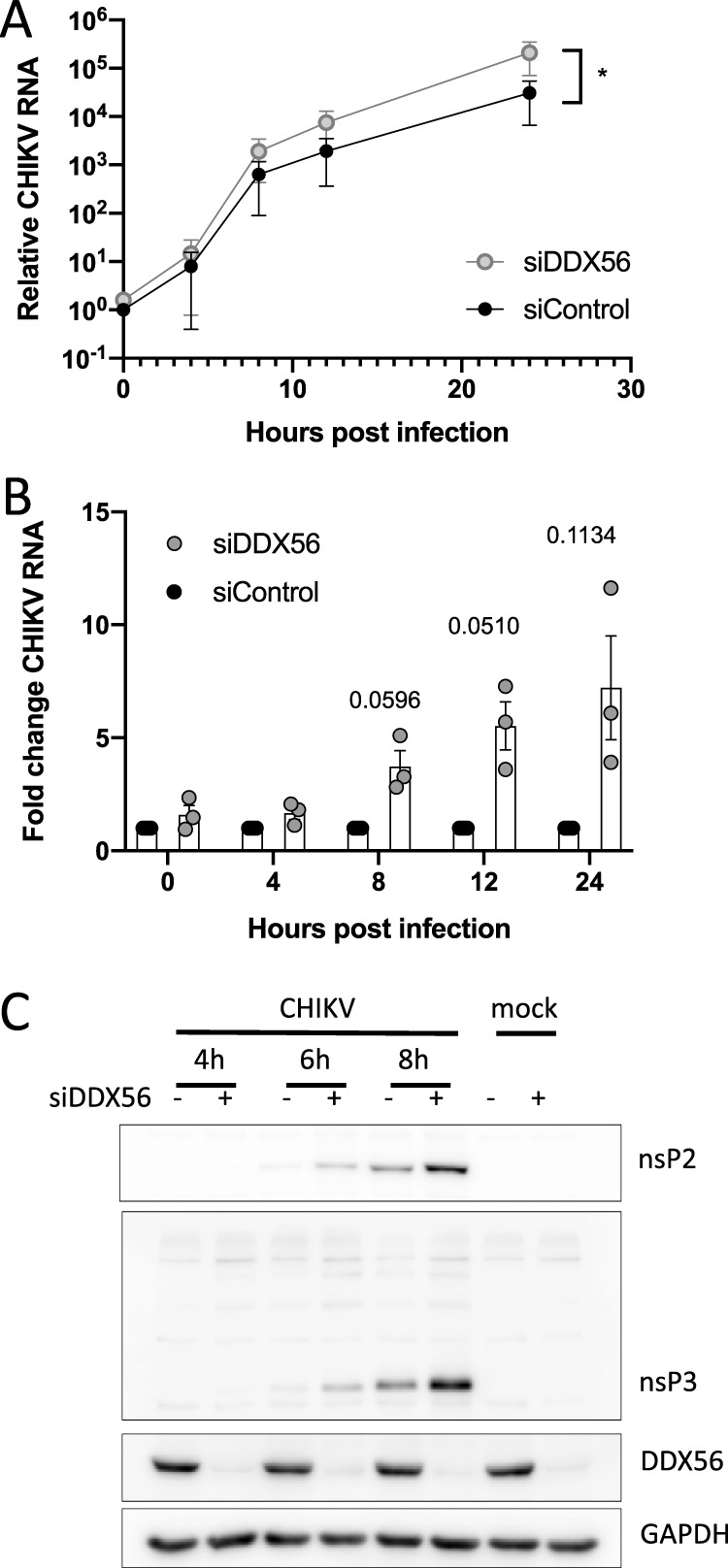
DDX56 controls an early step in CHIKV infection. (A and B) U2OS cells were transfected with control or DDX56 siRNAs and infected with CHIKV. Total RNA was collected at the indicated time points and quantified by RT-qPCR with a GAPDH control. (A) CHIKV RNA levels normalized to siControl-treated cells at 0 hpi to show increase in RNA levels over the course of infection. *P* < 0.05 by paired *t* test on ΔΔ*C_T_* values. (B) Data from panel A displayed as fold difference between DDX56-depleted cells and control cells at each time point. Statistical significance was assessed by one-sample *t* test on log-transformed fold change for each time point. Values are means and SEM, *n* = 3. (C) U2OS cells were transfected with control or DDX56 siRNAs and infected with CHIKV. Total protein lysates were collected at the indicated time points and analyzed by immunoblotting for CHIKV nsP2 and nsP3, DDX56 to show depletion, and GAPDH as a loading control. Representative experiments are shown, *n* ≥ 3. See also Fig. S3.

The location of the CLIP peak within the nonstructural open reading frame and adjacent to a region required for efficient nsp4 (RdRp) translation suggests that DDX56 binding impacts early steps in the replication cycle, as translation of nonstructural proteins is required for initial viral RNA synthesis. To determine how early in infection the DDX56 antiviral phenotype became apparent, we measured viral RNA levels over time in synchronized infections. CHIKV was prebound to cells at 4°C, and unbound virions were removed (*t* = 0) before shifting cells to 37°C to allow infection to proceed. We collected RNA every 4 h from control and DDX56-depleted cells and quantified viral RNA by RT-qPCR. Levels of viral RNA were similar in control and DDX56-depleted cells at *t* = 0 and *t* = 4 h, suggesting that binding and entry are not impacted. The antiviral effect of DDX56 becomes evident between 4 and 8 h postinfection, which is the time frame when viral RNA synthesis is launching, and becomes more pronounced as replication proceeds (Fig. 4A and B display these data in two ways, first as RNA increases over time and second as fold differences at each time point). This suggested that DDX56 restricts replication soon after entry, leading us to further investigate effects on early steps of viral replication.

10.1128/mBio.02623-20.3 FIG S3 DDX56 impacts early steps in CHIKV infection. Densitometry quantification of Western blot band intensity from Fig. 3C is shown. Gel lanes were analyzed in ImageJ. Signal is normalized to GAPDH loading and expressed relative to band intensity of siControl at 8 h postinfection. Results are displayed as mean and SEM for *n* ≥ 3. Download FIG S3, PDF file, 0.1 MB. Copyright © 2020 Taschuk et al. 2020 Taschuk et al. This content is distributed under the terms of the Creative Commons Attribution 4.0 International license.

To explore the effect of DDX56 on early CHIKV protein production, we evaluated levels of CHIKV nonstructural proteins by Western blotting. We monitored levels of two nonstructural proteins, nsP2 and nsP3, from 4 to 8 hpi, and found that DDX56 depletion resulted in increased production of viral nsPs. This effect was apparent as soon as these proteins became detectable by Western blotting (∼6 h postinfection) (Fig. 4C and Fig. S3). Together, these data indicate that DDX56 affects viral replication at the earliest stages of infection.

### DDX56 impacts incoming CHIKV genome stability.

**FIG 5:**
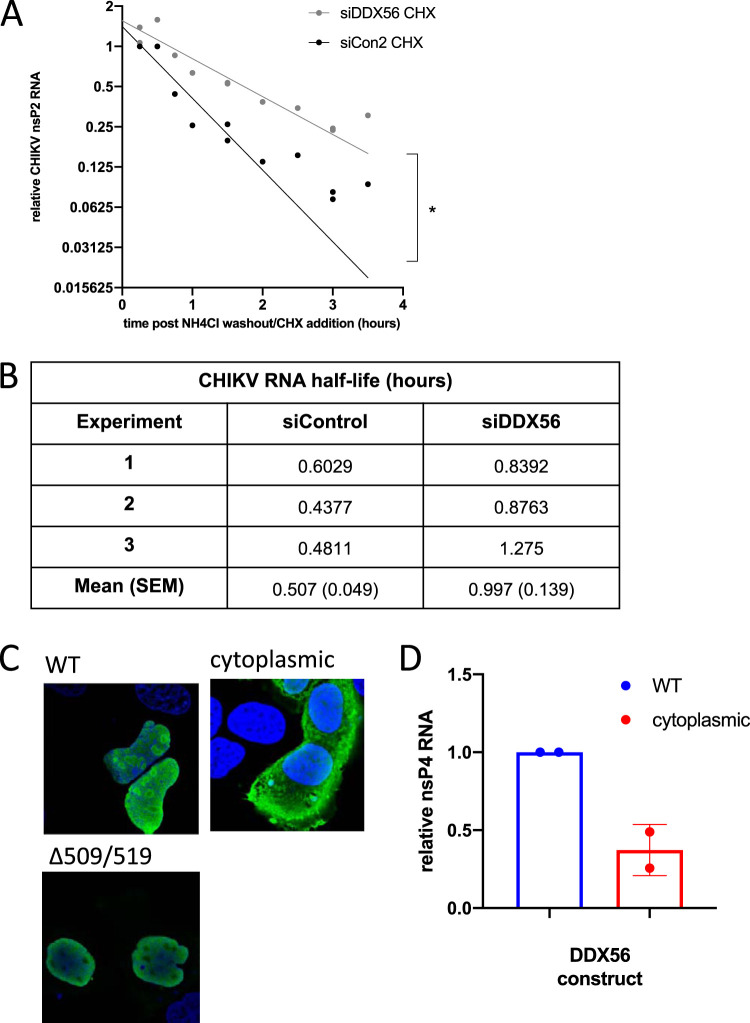
DDX56 destabilizes incoming viral genomic RNA. U2OS cells were transfected with control or DDX56 siRNAs, infected with CHIKV at 4°C to synchronize infection, washed, and treated with cycloheximide. Samples were collected at the indicated time points for RT-qPCR. (A) Compiled data from three experiments with exponential decay fit. (B) Half-lives for CHIKV RNA were calculated from exponential decay curves for three independent experiments. (C) U2OS cells were transfected with DDX56-HA expression vectors containing either a wild-type construct or mutations promoting cytoplasmic localization. Cells were stained with anti-HA and imaged by confocal microscopy. (D) DDX56 constructs were cotransfected with a CHIKV nsP4 reporter plasmid. Expression of CHIKV nsP4 from the reporter was quantified by RT-qPCR (*n* = 2).

Once CHIKV enters and uncoats in the cytoplasm, the incoming RNA genome must be translated to launch infection. Because we observed decreases in viral RNA production and in the percentage of cells that were productively infected (Fig. 1), as well as defects in viral protein levels at the earliest time points (Fig. 4C and Fig. S4), we reasoned that these findings might be explained by DDX56 binding to incoming genomes and attenuating the launch of viral replication. To determine whether DDX56 indeed affects the stability of incoming CHIKV RNA, we synchronized infection and monitored levels of viral RNA over time while blocking translation using cycloheximide (CHX), which blocks alphavirus minus-strand synthesis (31). We found that the half-life of CHIKV genomic RNA is approximately 0.56 h, and that upon depletion of DDX56 the half-life is extended ∼2-fold compared to that of cellular housekeeping mRNAs (Fig. 5). These data suggest that cytoplasmic DDX56 destabilizes incoming CHIKV genomic RNA, which attenuates viral infection.

10.1128/mBio.02623-20.4 FIG S4 Multiple pathways impact CHIKV stability. (A) U2OS cells were transfected with the control or with siRNAs targeting RNA decay pathways, and viral RNA stability was analyzed as in Fig. 5A. *n* = 3. *, *P* < 0.05 by unpaired *t* test on half-life values from three independent experiments. (B) Schematic of DDX56 overexpression constructs. Download FIG S4, PDF file, 0.1 MB. Copyright © 2020 Taschuk et al. 2020 Taschuk et al. This content is distributed under the terms of the Creative Commons Attribution 4.0 International license.

There are various RNA decay pathways in cells. We performed initial studies to determine which RNA decay pathways may contribute to DDX56-mediated destabilization of CHIKV genomes. To this end, we tested three pathways: the 5′ to 3′ decay machinery (Xrn1), the 3′ to 5′ decay machinery (RNA exosome; Dis3 + Rrp6), and the nonsense decay machinery (Upf1) (32, 33). For these studies, we depleted key factors in each of these RNA decay pathways and determined the effect on CHIKV genome half-lives. We depleted the control, Xrn1, Upf1, or Dis3 + Rrp6 and found that depletion of each had a modest effect and that Upf1 had the greatest effect on the half-life of incoming CHIKV genomes (Fig S4A). This suggests complex regulation of genomic RNA decay.

### DDX56 localization affects its activity on CHIKV RNA.

DDX56 is a nucleolar resident protein that we found relocalizes during CHIKV infection. DDX56 contains lysine- and arginine-rich motifs in its C-terminal sequence, which have been identified as putative nuclear localization signals (NLS) (21). To investigate the importance of DDX56 localization to its interactions with CHIKV RNA and, therefore, its antiviral effect, we made a series of mutations in a hemagglutinin (HA)-tagged DDX56 construct. We changed multiple positively charged amino acids to alanine (509-KKRKK to AARAA and 519-RKAKR to AAAAA) in the putative nuclear localization signal. When this construct was expressed, it remained in the nucleus but was no longer localized to the nucleoli, as is the HA-tagged wild-type (WT) construct (Fig. 5C). It has been observed in other DEAD box helicases that nucleocytoplasmic shuttling can involve multiple nuclear localization sequences as well as nuclear export sequences (NES) (34). Therefore, to promote cytoplasmic expression of our construct, we added the NES from HIV-Rev to the C-terminal end of the double NLS mutant (DDX56-cyto), which resulted in cytoplasm-localized DDX56-HA expression (Fig. 5C and Fig. S4B).

To assess the importance of DDX56 localization for its effect on the CHIKV RNA stem-loop, we cotransfected WT or cytoplasm-localized DDX56 along with a reporter plasmid containing the CHIKV nsP4 gene that includes the stem-loop. The levels of the mRNA reporter were reduced in cells expressing cytoplasmic DDX56 compared to those of wild-type, nucleolar DDX56 (Fig. 5D). This demonstrates that the localization of DDX56 impacts its activity.

## DISCUSSION

DEAD box helicases play fundamental roles in all aspects of cellular RNA metabolism. This requires interaction of the DEAD box domain with distinct RNAs to carry out these diverse functions. Our knowledge of the interactions of DEAD box helicases with viral RNAs is incomplete. Given that these helicases bind structural features of RNAs and viral RNAs are known to be highly structured, we and others have begun to characterize these interactions. Our genetic screening identified three DEAD box RNA helicases, DDX56, DDX23, and DDX46, as antiviral against alphaviruses in both arthropod and human cells. We focused on DDX56, which we found is antiviral against both SINV and CHIKV early in infection. While DDX56 is predominantly nucleolar, a fraction is also present in the cytoplasm where viral RNA resides, and cytoplasmic DDX56 levels increase during the early phases of infection. This led us to discover that DDX56 directly binds cytoplasmic viral RNA. We found that DDX56 interaction with the incoming viral genome decreases the stability of viral RNA, preventing this RNA from efficient translation and launching of infection.

In addition to finding an interaction between DDX56 and CHIKV RNA, CLIP-Seq allowed us to determine specific features of this interaction. First, we found a dearth of binding to the negative-sense antigenome. While this viral RNA species is indeed the least prevalent, the conspicuous lack of signal suggests that this RNA is protected from RNA binding proteins, and it has been shown that Semliki Forest virus (SFV) antigenomes are sequestered in membrane spherules capable of protecting them from experimental RNase treatment (35). Second, we found that sequences across the subgenomic RNA were bound by DDX56. This is the most prevalent viral mRNA in infected cells at the time point we investigated by CLIP-Seq, but the distribution across this mRNA suggests interactions that have low specificity. Third, and most strikingly, we found a strong peak on the genomic RNA adjacent to a previously characterized stem-loop (17, 22). This stem-loop feature is conserved across alphaviruses, including CHIKV and SINV, and promotes translation of the RdRp nsP4 early in infection. Translation of nonstructural proteins from the incoming viral genome is required to launch RNA replication and transcription of the subgenomic RNA. That DDX56 binds a potentially structured region adjacent to this stem-loop suggests that this region of the genome is of particular importance for regulating early steps of infection. Future studies are needed to define the features required for DDX56 binding in this region.

We found that DDX56 impacts the stability of the incoming viral genome, with CHIKV RNA exhibiting a longer half-life in cells depleted for DDX56. Furthermore, overexpression of a cytoplasmic-localized DDX56 construct resulted in lower levels of CHIKV nsP4 mRNA expressed from a reporter. Taken together with our observations of increased cytoplasmic localization of DDX56 during CHIKV infection and our CLIP-Seq findings that DDX56 binds specifically to CHIKV RNA in the nsP4 gene, we hypothesize a model where DDX56 in the cytoplasm interacts with the nsP4 region of the CHIKV genome, leading to destabilization of the viral RNA. Because a fraction of DDX56 is present in the cytoplasm at baseline, this interaction may begin as soon as CHIKV enters a cell and uncoats. However, accumulation of DDX56 in the cytoplasm over the course of CHIKV infection suggests that as infection proceeds, more DDX56 becomes available for cytoplasmic interaction with viral RNA.

Viruses must evade RNA degradation pathways or coopt stabilizing factors in order to replicate. Stability factors such as HuR can interact directly with viral RNA and promote replication, including binding the 3′ untranslated region of SINV (36). Conversely, mRNA degradation pathways are known to limit infection by various RNA viruses. Decapping and 5′ to 3′ exonucleocytic degradation limits bunyavirus and flavivirus infections, with the decapping activator helicase DDX6 identified as an antiviral factor in both cases (37, 38). The RNA exosome pathway, including the DExD/H RNA helicase MTR4, has been found to be antiviral against the alphavirus SINV, the bunyavirus RVFV, and the vesiculovirus VSV (33, 39). Nonsense-mediated decay is also important for restriction of RNA viruses, including the flavivirus Zika virus (40), the coronavirus mouse hepatitis virus (41), and the tombusvirus turnip crinkle virus (42). The NMD factor UPF1 has been shown to limit early cytoplasmic replication steps of the alphaviruses SFV and SINV (39). Our observation that UPF1 destabilizes CHIKV RNA upon entry provides further evidence for the importance of NMD in alphavirus infection.

When DEAD box helicases bind RNA, they can serve as platforms for recruiting additional effectors or driving assembly of ribonucleoprotein complexes to promote RNA degradation. DDX17 (P72) serves as a cofactor for ZAP to promote degradation of viral sequences by the exosome and also interacts with the exosome in an RNA-dependent manner in the absence of ZAP (43). For spliced cellular mRNAs, binding of the DEAD box helicase EIF4A3 to mRNA as part of the pre-exon junction complex contributes to recruitment of NMD-activating factors and leads to UPF1-dependent degradation (44). Helicases can also drive accumulation of larger RNP condensates; DDX6 is essential for formation and maintenance of RNA processing bodies (P-bodies), where it promotes mRNA decapping and 5′ to 3′ degradation via its interactions with proteins of the decapping complex (45). Our data suggest a model in which DDX56 recruits RNA decay machinery to destabilize viral RNA, possibly through direct or indirect interactions with UPF1. Future studies will address how DDX56 and UPF1 act together to modulate viral RNA levels and whether cellular functions of DDX56 are likewise associated with nonsense-mediated decay or other RNA degradation pathways.

While DDX56 is largely nucleolar, consistent with its role in rRNA biogenesis, we found that a subset of DDX56 accumulates in the cytoplasm upon CHIKV infection. Viral infection is known to impact many aspects of cellular function, and we and others have observed that localization of RNA-binding proteins and nucleolar factors is disrupted during alphavirus infection, including DDX56, nucleolin, NPM1, and fibrillarin (46 and data not shown). Therefore, changes in the localization of RNA binding proteins, including DDX56, may be a part of a larger program in cells. Other viruses also relocalize DDX56; a previous study found that during West Nile virus infection, DDX56 relocalizes to the cytoplasm to colocalize with viral assembly sites and is required for efficient viral RNA packaging (47, 48). Interestingly, we observed neither DDX56 accumulation in the cytoplasm nor nucleolar disruption in cells infected with dengue virus, suggesting that this phenomenon is specific to particular viral infections (data not shown).

Our studies also found that two other nuclear helicases, DDX23 and DDX46, were antiviral in both insect and human cells. DDX23 and DDX46 are part of the core splicing machinery (49). A recent study found that DDX23 is antiviral against dengue virus, and dengue virus NS5 interacts with DDX23 to facilitate infection via modulation of spliceosome activity (50). Whether DDX23 or DDX46 is antiviral against alphaviruses through modulation of splicing is unknown.

Overall, our data describe a novel role for DDX56 as an antiviral factor. Binding of viral RNA by this host protein as it enters the cytoplasm leads to the destabilization and loss of the infectious RNA. Since infections *in vivo* are thought to be at low multiplicities of infection, the presence of DDX56 increases the likelihood that an incoming viral genome will be degraded rather than translated. Thus, DDX56 targeting of viral RNA may lead to clearance of infection from a cell prior to the launch of viral replication. Future studies will investigate whether we can manipulate this pathway by increasing the levels of cytoplasmic DDX56 or increasing the efficiency of viral RNA degradation to enhance cellular defenses against alphavirus infection.

## MATERIALS AND METHODS

### Cells, viruses, and reagents.

Drosophila melanogaster DL1 cells were obtained from DRSC and maintained at 25°C in Schneider’s *Drosophila* medium (Gibco) containing 10% fetal bovine serum (FBS), l-glutamine (Gibco), and penicillin-streptomycin (Gibco). U2OS cells (HTB-96; RRID:CVCL_0042) and HeLa cells were obtained from ATCC and maintained at 37°C in Dulbecco’s modified Eagle’s medium (DMEM) (Gibco) supplemented with 10% FBS, l-glutamine (Gibco), and penicillin-streptomycin (Gibco).

CHIKV isolate PC-08 (51) was obtained from D. Weiner (Wistar Institute). CHIKV-mKate (181/25) was obtained from M. Heise (University of North Carolina). SINV-GFP was from R. Hardy (University of Indiana). VSV-enhanced GFP (VSV-eGFP) was a gift from J. Rose (Yale). Viruses were propagated in C636 cells and titers determined on BHK cells, except VSV-eGFP was grown in BHK cells as described previously (52). Viral strains were handled at biosafety level 2.

### siRNA transfection.

siRNAs for DDX56 and controls were obtained from Ambion (SilencerSelect) or Sigma (sequences are in Table S3 in the supplemental material). AllStars Hs Cell Death siRNA (1027299; Qiagen) was used to verify knockdown in each experiment, and specific knockdowns were compared to a nontargeting control siRNA (AM4613; Life Technologies). U2OS cells were transfected using HiPerFect reagent (301709; Qiagen) with a final siRNA concentration of 20 nM by following the manufacturer’s protocol. Two pooled siRNAs were used for DDX56 experiments, but we validated that each could independently knock down DDX56 and affect CHIKV infection. Cells were infected on day 3 posttransfection and collected at the indicated time points postinfection.

10.1128/mBio.02623-20.7 TABLE S3 Oligonucleotides used in this study. Detailed information for oligonucleotides used in siRNA knockdown, CLIP-Seq, and PCR amplification is given. Download Table S3, DOCX file, 0.1 MB. Copyright © 2020 Taschuk et al. 2020 Taschuk et al. This content is distributed under the terms of the Creative Commons Attribution 4.0 International license.

### Viral infections.

To quantify the effect of DDX56 on viral infection, cells were infected with SINV (multiplicity of infection [MOI], 0.1 to 0.3) and collected 16 to 20 hpi, with CHIKV (MOI, 0.1 to 0.3) and collected 20 to 24 hpi, and with VSV (MOI, 0.25 to 1) and collected 10 hpi. Infections were monitored by microscopy or RT-qPCR assays. For biochemical and sequencing studies, cells were infected with CHIKV at an MOI of 5.

At the indicated time points, cells were washed once with phosphate-buffered saline (PBS) and collected in TRIzol (15596; Thermo) for RNA extraction according to the manufacturer’s instructions or fixed with 4% formaldehyde for 10 min for quantification by microscopy. Cells were stained for immunofluorescence imaging using anti-CHIKV ascites V-548-701-562 (ATCC VR-1241AF) at 1:300 or CHK-265 (gift from M. Diamond) at 1:500 and anti-mouse 488 and Hoechst 33342 (B2261; Sigma-Aldrich) at a final concentration of 5 μg/ml. SINV-GFP and VSV-GFP reporter viruses were counterstained with Hoechst. Automated imaging and image analysis (ImageXpress Micro; Molecular Devices) were used to quantify cell number and percent infection where 3 sites per well were averaged, and at least 3 independent experiments were used for statistical analysis using *t* tests.

### Viral RNA time courses.

For CHIKV replication time courses, U2OS cells were infected at an MOI of 10, and infection was synchronized by allowing viruses to bind to cells at 4°C for 1 h. Cells were washed twice with PBS to remove unbound virus, and plates were transferred to 37°C in complete culture medium. At the indicated time points, samples were washed once with PBS and collected in TRIzol. RNA extraction was performed using a column-based kit (R1018; Zymo Research) with in-column DNase treatment (E1010; Zymo Research).

To assess stability of entering viral genomes, U2OS cultures were infected at an MOI of 50; infection was synchronized by allowing viruses to bind to cells at 4°C for 1 h, washing twice with PBS to remove unbound virus, and transferring cells to 37°C with complete medium containing cycloheximide at 20 μg/ml (1988; Sigma). At each time point, cells were digested with 0.25% trypsin to remove surface-bound virions and washed once with PBS before collection in TRIzol (15596018; Life Technologies) for RNA extraction using a column-based kit (R1018; Zymo Research) with in-column DNase treatment (E1010; Zymo Research). Viral RNA half-life was calculated by nonlinear regression, and half-lives from 3 experiments were compared using an unpaired two-tailed *t* test.

### Reverse transcription and qPCR.

RNA samples were reverse transcribed using MMLV RT (28025013; Thermo) and random hexamers according to the manufacturer’s instructions. Diluted cDNA was used for SYBR-based qPCR (4368577; Applied Biosystems). Relative quantification was calculated according to the ΔΔ*C_T_* method (53). Significance was measured using a one-sample *t* test on log_2_(fold change). Primer sequences are listed in Table S3.

### Immunoblot analysis.

Cells were collected by trypsinization and lysed in radioimmunoprecipitation assay (RIPA) buffer supplemented with protease inhibitors. Samples were run on Tris-glycine SDS-PAGE gels and transferred to polyvinylidene difluoride (PVDF) membranes (Immobilon) for Western blot detection. Blots were exposed with chemiluminescent regents and imaged with a charge-coupled device (CCD)-based detection system (Amersham). The following antibodies were used: DDX56 (A302-979A, RRID:AB_10750607; Bethyl), glyceraldehyde-3-phosphate dehydrogenase (GAPDH; CB1001, RRID:AB_2107426; Millipore), lamin B1 (ab16048, RRID:AB_443298; Abcam), tubulin (T6199, RRID:AB_477583; Sigma-Aldrich), CHIKV nsP1 (11–13020; Abgenex), CHIKV nsP2 (10–10024; Abgenex), and CHIKV nsP3 (11–13022; Abgenex).

### Nuclear-cytoplasmic fractionation.

Fractionation was performed as previously described (33). Cellular membranes were lysed by needle shearing in 30 mM HEPES, pH 7.4, 2 mM MgOAc, 0.1% NP-40, protease inhibitors (Roche), and phenylmethylsulfonyl fluoride. Nuclei were pelleted by centrifugation, cytoplasmic extracts were removed, and nuclei were washed with lysis buffer and lysed in RIPA buffer by sonication at amplitude 5, three times for 10 s each time with 10-s pauses. Samples were analyzed by immunoblotting.

### CLIP-Seq.

CLIP-Seq was performed with a protocol modified from Vourekas and Mourelatos (26). U2OS cells were plated at 10E6 per 15-cm dish and infected with CHIKV (MOI, 5). At 20 h postinfection, cells were trypsinized, cross-linked in a UV Stratalinker at 400 mJ/cm^2^, and frozen at −80°C. Cell lysates were prepared in a DNase-PMPG buffer and were RNase digested to leave RNA fragments of an appropriate size for sequencing. Samples were immunoprecipitated using anti-DDX56 (A302-979A; Bethyl) or rabbit IgG control (P120-201; Bethyl) antibodies bound to Dynabeads protein A beads (10002D; Thermo). A ^32^P-labeled RNA adapter was ligated to the 3′ end of RNA, and samples were run on an SDS-PAGE gel and transferred to nitrocellulose membrane. ^32^P signal from the adapter-labeled RNA-protein complexes was used as a guide to excise appropriate regions of the membrane and extract RNA. A 5′ RNA adapter containing a degenerate barcode was ligated to the extracted RNA. Samples were reverse transcribed and PCR amplified to prepare the sequencing library, using primers that added specific index sequences for each sample to allow multiplexing of sequencing runs. Samples were pooled and sequenced on Illumina HiSeq at the University of Pennsylvania Next Generation Sequencing Core. Results represent three independent sets of infections and immunoprecipitations.

### Next-generation sequencing data analysis.

Raw FastQ reads were trimmed using cutadapt (54) and Trimmomatic (55). Unique molecular identifier barcodes were removed and exact duplicate sequences within each file were collapsed using the CLIP Tool Kit fastq2collapse (28). Reads were aligned to a composite genome consisting of hg19 (including unlocated contigs) with the CHIKV genomic sequence (Ross strain; GenBank accession no. AF490259.3) appended and treated as an additional chromosome. To exclude cross-contaminating signal from our analysis of viral reads, reads aligning to the CHIKV genome were further filtered to exclude any reads that matched in sequence, barcode, and mapping location between samples within each replicate. CLIP Tool Kit tag2collapse was then used to collapse PCR duplicates that mapped to the same genomic coordinates. Analyses were run on the Penn Medicine Academic Computing Services High-Performance Computing System, and results were visualized in R and IGV. Figures were generated in R. Software packages used are listed in Table S4. CLIP-Seq read coverage on the CHIKV genome can be found in Tables S1 and S2 for CHIKV_coverage_plus.xlsx and CHIKV_coverage_minus.xlsx.

10.1128/mBio.02623-20.8 TABLE S4 Analysis and visualization software. List of software tools and R packages used in data analysis and figure generation; includes references and version information. Download Table S4, DOCX file, 0.1 MB. Copyright © 2020 Taschuk et al. 2020 Taschuk et al. This content is distributed under the terms of the Creative Commons Attribution 4.0 International license.

### Cloning and plasmid transfection.

A cDNA clone of DDX56 (accession no. BC001235) was obtained from the MGC ORFeome collection. An HA tag was added to the C-terminal end of the DDX56 open reading frame by PCR amplification using Phusion polymerase and recircularization using T4 DNA ligase. Site-directed mutagenesis of the DDX56 cDNA sequence was performed in the same way. A cGFP-nsP4-Flag reporter construct was constructed by inserting nsP4-Flag amplified from CHIKV replicon pJM40 (strain SL15649; courtesy of Mark Heise, UNC) into the plasmid pCRII-TOPO CMV-cGFP-SV40 (56) between cGFP and the simian virus 40 poly(A) site using a NEBuilder HiFi cloning kit (NEB). Cloning primers are listed in Table S3.

Plasmids were transfected into U2OS cells by electroporation in 4-mm cuvettes using a Bio-Rad GenePulser at 210 V and 960 mA. For cotransfection experiments, DDX56 and reporter constructs were used in a 3:1 ratio by mass. Expression was analyzed at 1 to 2 days postelectroporation by RNA extraction, reverse transcription, and qPCR as described above.

### RNA folding predictions.

Secondary structures of viral RNA sequences were predicted using default parameters for RNAfold on the Vienna RNA webserver based on Vienna version 2.4.13 (30). Conservation at base-pairing positions was visualized for CHIKV strains representing all major lineages (accession numbers HM045811, HM045793, HM045795, HM045809, KT327167, KJ451624, KX262994, KX262992, HM045787, EF452493, HM045801, KY575571, MK028837, and HM045798) (57) using the R4RNA R package version 1.10.0 (58). Figures were generated using R4RNA and VARNA version 3.9 (59). Regions modeled correspond to coordinates 5658 to 5852 of CHIKV (accession number AF490259.3) and 5763 to 6019 of SINV hrsp (accession number J02363).
