# Supplementary figures and images for: DDX56 Binds to Chikungunya Virus RNA To Control Infection
Source: mBio. 2020 Oct 27;11(5):e02623-20. doi: 10.1128/mBio.02623-20 (PMC7593974; doi:10.1128/mBio.02623-20)

Figure S1

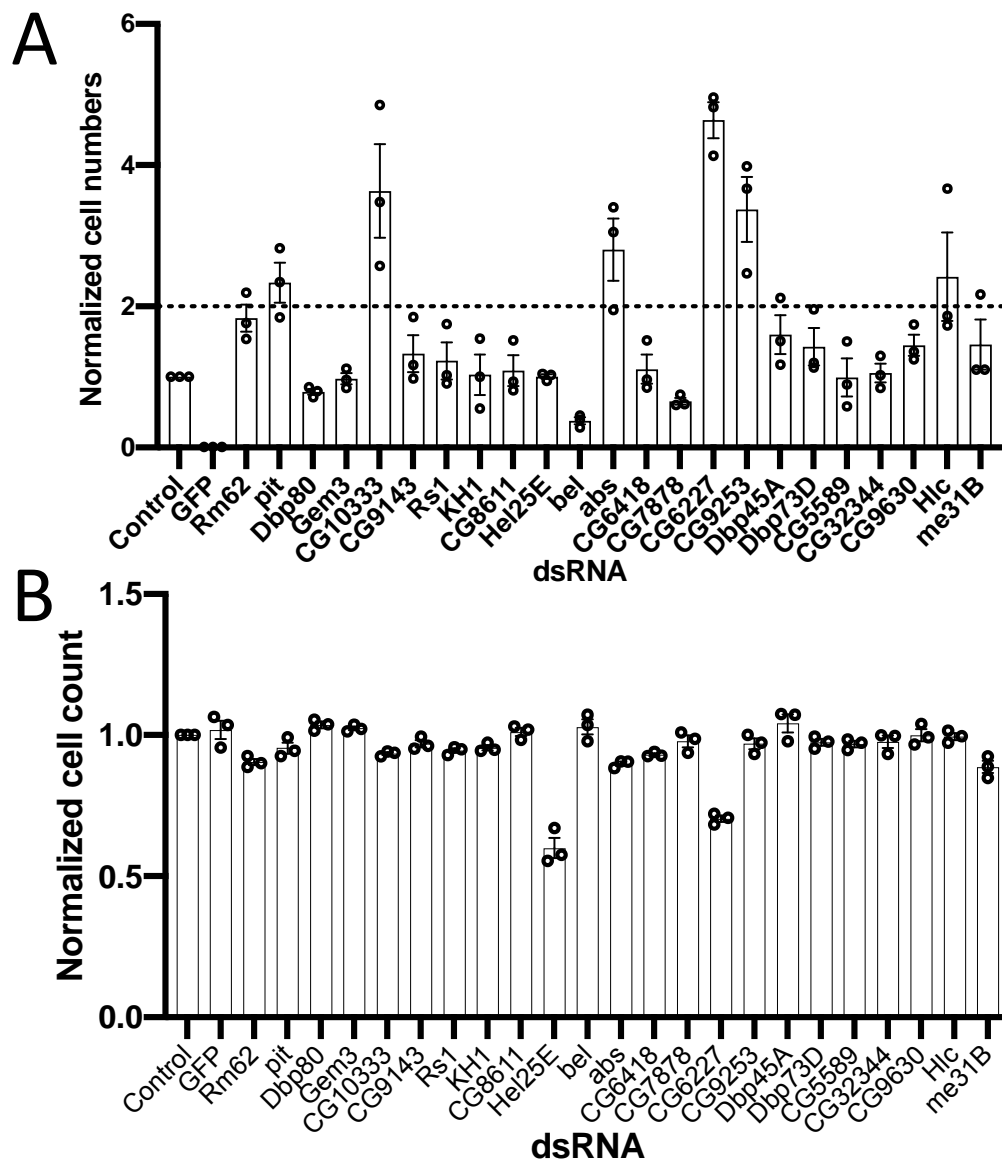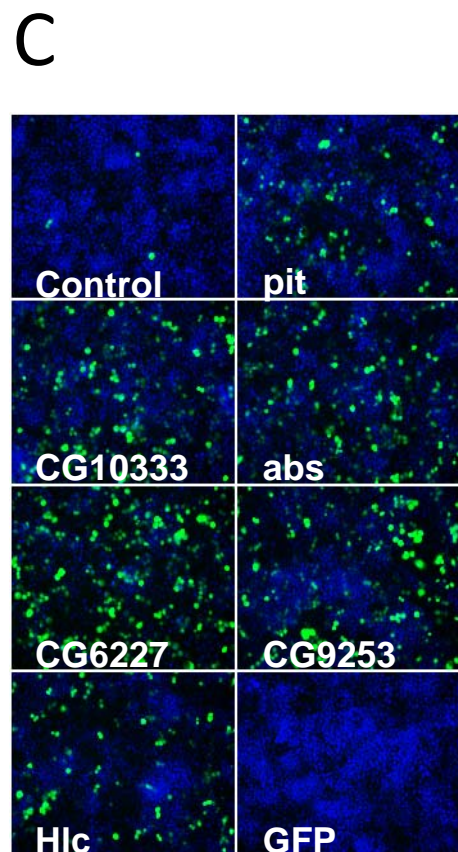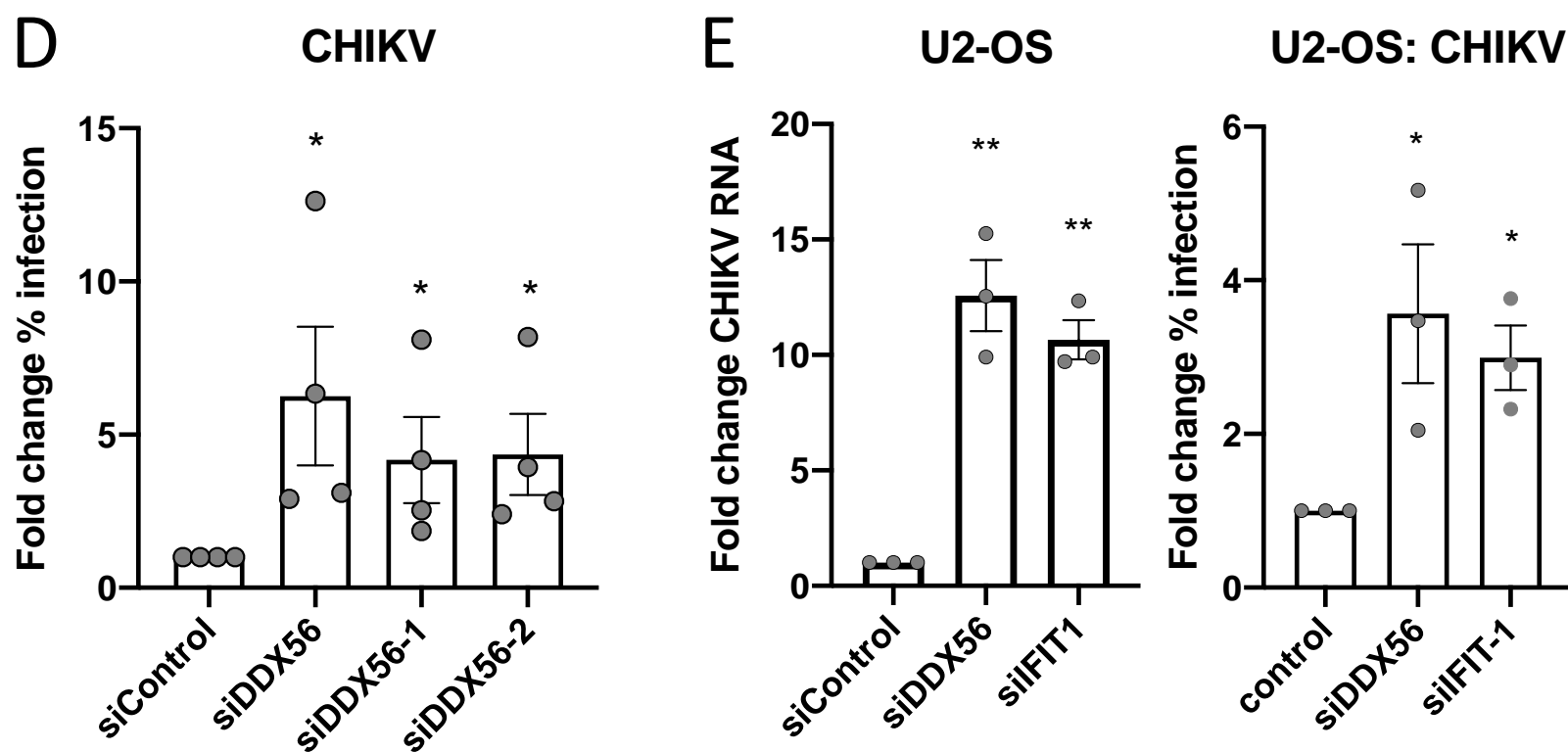

Supplement: FIG S1 [file mBio.02623-20-sf001.pdf]

Figure S3

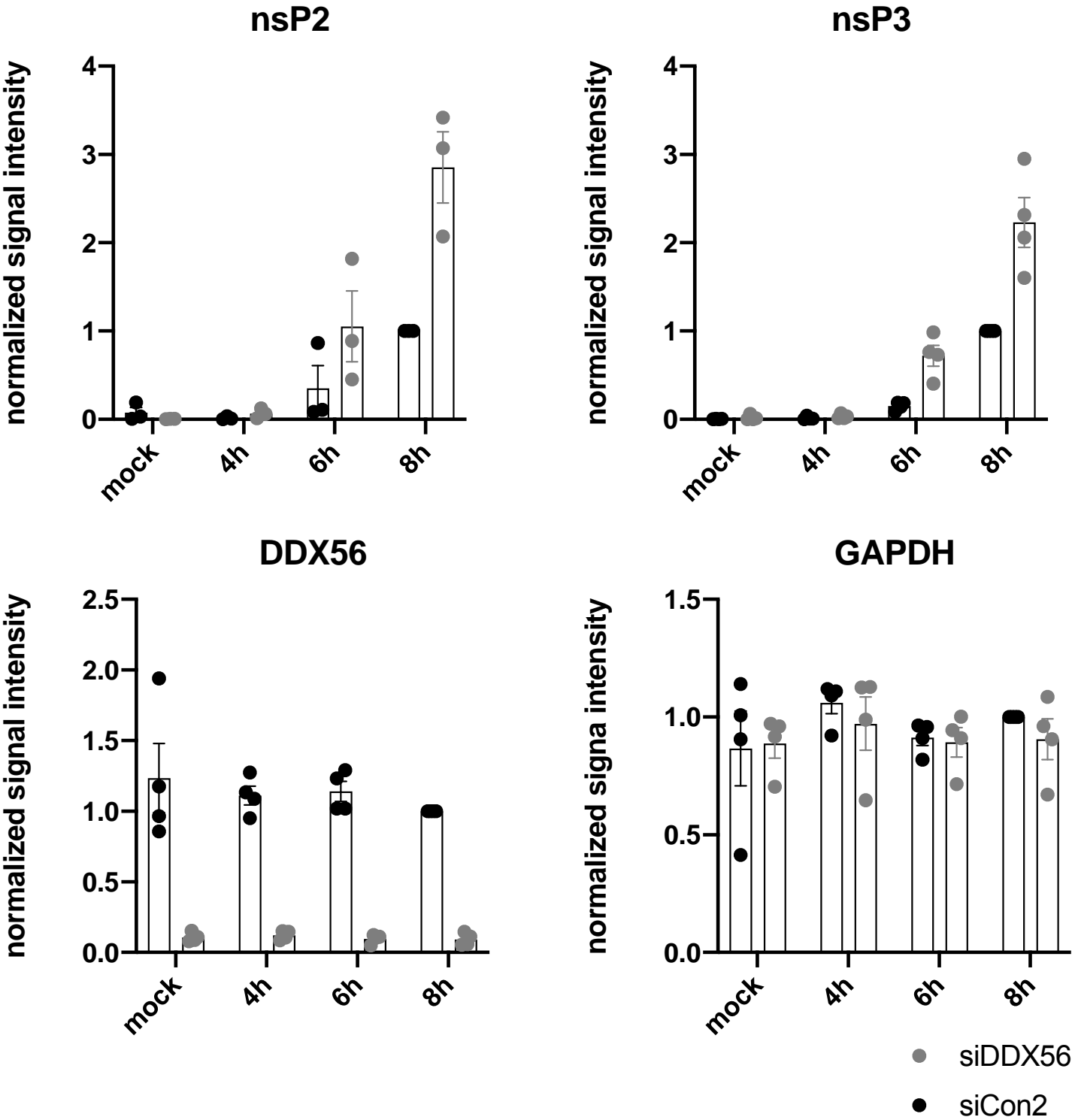

Supplement: FIG S3 [file mBio.02623-20-sf003.pdf]

Figure S4

A

Compiled NH4Cl synch - quantification - control vs decay

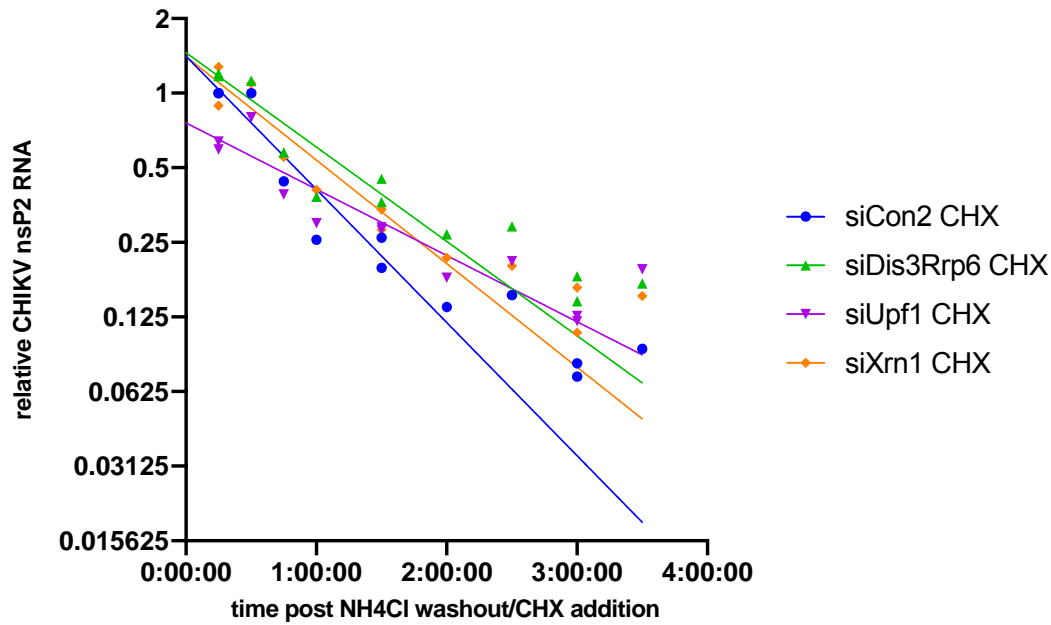

B

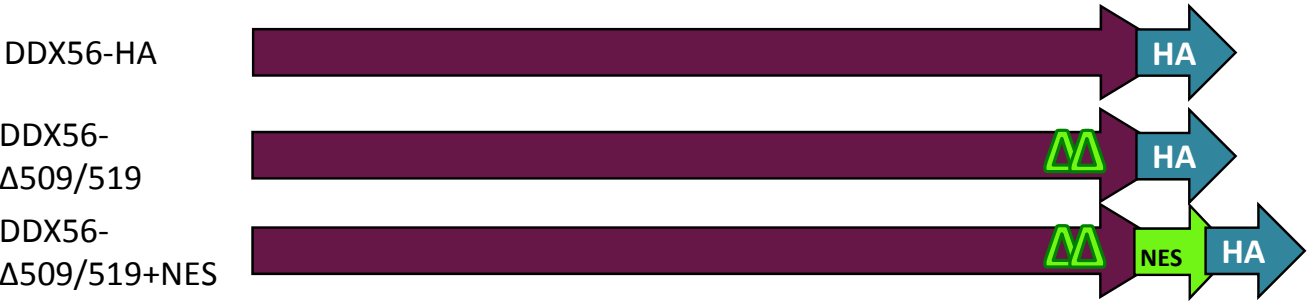

Supplement: FIG S4 [file mBio.02623-20-sf004.pdf]
