# Supplementary material for: DDX56 Binds to Chikungunya Virus RNA To Control Infection
Source: mBio. 2020 Oct 27;11(5):e02623-20. doi: 10.1128/mBio.02623-20 (PMC7593974; doi:10.1128/mBio.02623-20)
Supplement: FIG S2 [file mBio.02623-20-sf002.pdf]

Figure S2

A

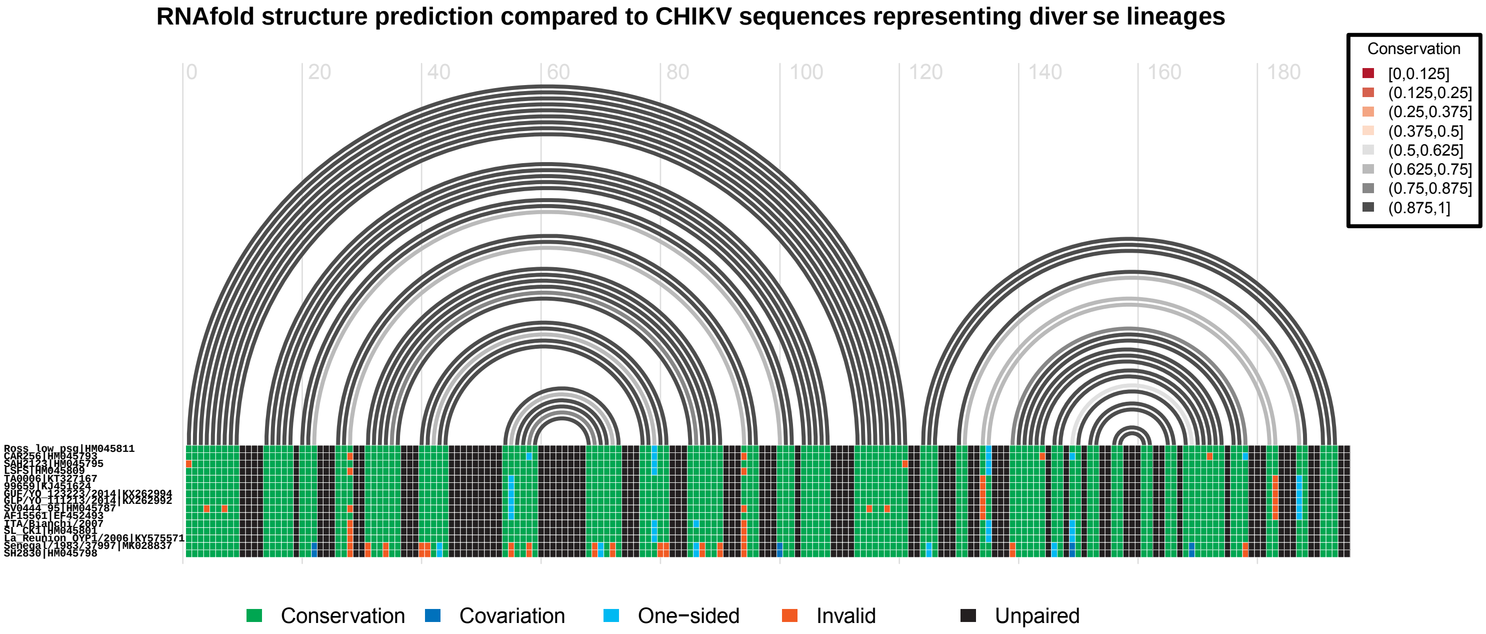

RNAfold structure prediction compared to CHIKV sequences representing diver se lineages
0
20
40
60
80
100
120
140
160
180
Conservation
[0,0.125]
(0.125,0.25]
(0.25,0.375]
(0.375,0.5]
(0.5,0.625]
(0.625,0.75]
(0.75,0.875]
(0.875,1]
Ross_low_psg|HM045811
CAR256|HM045793
SAH2123|HM045795
LSFS|HM045809
TA0006|KT327167
99659|KJ451624
GUF/YO_123223/2014|KX262994
GLP/YO_111213/2014|KX262992
SV0444_95|HM045787
AF15561|EF452493
ITA/Bianchi/2007
SL_CK1|HM045801
La_Reunion_OYP1/2006|KY575571
Senegal/1983/37997|MK028837
SH2830|HM045798
Conservation
Covariation
One−sided
Invalid
Unpaired

B

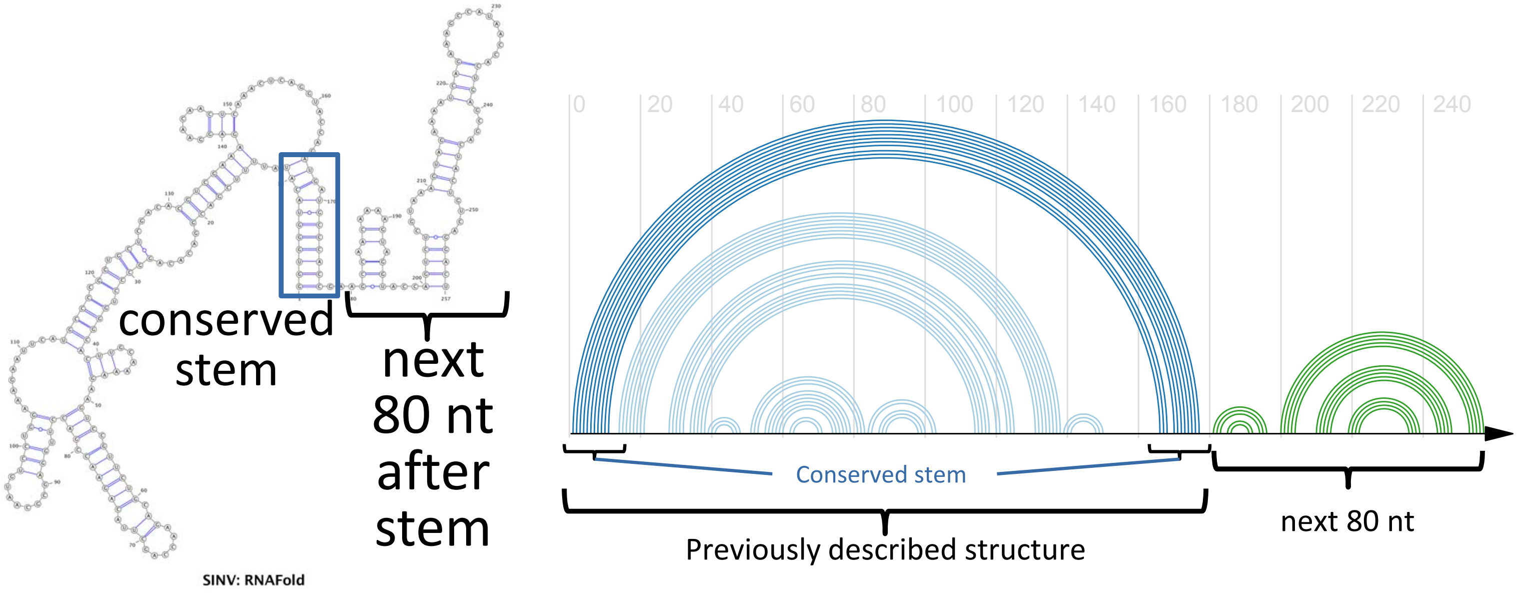

conserved stem
next 80 nt after stem
SINV: RNAFold
0
20
40
60
80
100
120
140
160
180
200
220
240
Conserved stem
Previously described structure
next 80 nt
