# Supplementary material for: DDX56 Binds to Chikungunya Virus RNA To Control Infection
Source: mBio. 2020 Oct 27;11(5):e02623-20. doi: 10.1128/mBio.02623-20 (PMC7593974; doi:10.1128/mBio.02623-20)
Supplement: TABLE S3 [file mBio.02623-20-st003.docx]

| **Table S3. Oligonucleotides used in this study** | | | |
| --- | --- | --- | --- |
| **siRNAs** | | | |
| **OLIGO** | **SOURCE** | **IDENTIFIER/SEQUENCE** | |
| siControl | Life technologies | #AM4613 | |
| siDeath | Qiagen | Allstars Hs Cell Death siRNA #1027299 | |
| siDDX56-1 | Sigma | sense: cccgguuacccuuaaguua[dT][dT]  antisense: uaacuuaaggguaaccggg[dT][dT] | |
| siDDX56-2 | Sigma | sense: gcuucaagcacaaaggaaa[dT][dT]  antisense: uuuccuuugugcuugaagc[dT][dT] | |
| **Oligos used in CLIP-Seq** | | | |
| **OLIGO** | **SOURCE** | **IDENTIFIER/SEQUENCE** | **REF.** |
| RL5D | IDT | /5InvddT/rArGrGrGrArGrGrArCrGrArUrGrCrGrGr(N1:25252525)r(N1)r(N1)r(N1)rG | (1) |
| RL3(+P) | IDT | /5Phos/rGrUrGrUrCrArGrUrCrArCrUrUrCrCrArGrCrGrG/3InvdT/ | (1) |
| RL3(-P) | IDT | rGrUrGrUrCrArGrUrCrArCrUrUrCrCrArGrCrGrG/3InvdT/ | (1) |
| P3-*C*A*C | IDT | CCGCTGGAAGTGACTGA*C*A*C | (1) |
| DP5 | IDT | AGGGAGGACGATGCGG | (1) |
| MSFP3 | IDT | CAAGCAGAAGACGGCATACGAGATCCGCTGGAAGTGACTGACAC | (1) |
| Index primers | IDT | AATGATACGGCGACCACCGAGATCTACACTCTTTCCCTACACGACGCTCTTCCGATCT[index]AGGGAGGACGATGCGG | (1) |
|  |  | (index sequences: CTAG,GATC,CGTA,GCAT,GTGC,ACCG,CATG,TGCA,TCAC,AGTG,TACG,CCGA) |  |
| **PCR primer sets** | | | |
| **TARGET** | **SOURCE** | **IDENTIFIER/SEQUENCE** | **REF.** |
| CHIKV nsP2 | IDT | forward: ggcagtggtcccagataattcaag;  reverse actgtctagatccaccccatacatg | modified from (2) |
| DDX56 | IDT | forward: ggcatgacctacctttgcac; reverse: ttcttccgcttcttgtgagg |  |
| GAPDH | IDT | forward: accaaatccgttgactccgacctt;  reverse: tcgacagtcagccgcatcttcttt | (3) |
| 18s rRNA | IDT | forward: aacccgttgaaccccatt; reverse: ccatccaatcggtagtagcg | (3) |
| SINV nsP1 | IDT | forward: gctgaaacaccatcgctctgcttt;  reverse: tggtgtcgaagccaatccagtaca | (3) |
| DDX56 cloning: C-terminal HA tag | IDT | forward: gccggattatgcgTGAGGTTGTTGGGCCTCTCTG;  reverse: acatcatacggataCATGGCGCTGCTCAGTAG |  |
| DDX56 cloning: mutate first putative NLS | IDT | forward: ggccgccCTGTCTTCCTCTTGTAGGAAGGCCAAG; reverse: cgggcggcGTGAGGGCGCACCAGGCC |  |
| DDX56 cloning: mutate second putative NLS | IDT | forward: cgccgccGCAAAGTCCCAGAACCCA;  reverse: gcggcggcACAAGAGGAAGACAGCTTC |  |
| DDX56 cloning: add NES | IDT | forward: gagagacttactcttgatTATCCGTATGATGTGCCGGATTATG; reverse: aagcggtggtagctgaagGGAGGGCTTGGCTGTGGG |  |
| AcGFP vector | IDT | forward: TGAGCGGCCGCAACTTGT;  reverse: CTTGTACAGCTCGTCCATG |  |
| nsP4-Flag | IDT | forward: gcatggacgagctgtacaagCGACTAGACAGGGCAGGTG; reverse: aaacaagttgcggccgctcactacttgtcatcgtcatccttg |  |

References:

1. Vourekas A, Mourelatos Z. 2014. HITS-CLIP (CLIP-Seq) for Mouse Piwi Proteins, p 73-95. *In* Siomi MC (ed) doi:10.1007/978-1-62703-694-8_7. Humana Press, Totowa, NJ.

2. Ho PS, Ng MM, Chu JJ. 2010. Establishment of one-step SYBR green-based real time-PCR assay for rapid detection and quantification of chikungunya virus infection. Virol J 7:13.

3. Molleston JM, Sabin LR, Moy RH, Menghani SV, Rausch K, Gordesky-Gold B, Hopkins KC, Zhou R, Jensen TH, Wilusz JE, Cherry S. 2016. A conserved virus-induced cytoplasmic TRAMP-like complex recruits the exosome to target viral RNA for degradation. Genes Dev 30:1658-70.
