# Supplementary material for: DDX56 Binds to Chikungunya Virus RNA To Control Infection
Source: mBio. 2020 Oct 27;11(5):e02623-20. doi: 10.1128/mBio.02623-20 (PMC7593974; doi:10.1128/mBio.02623-20)
Supplement: TABLE S4 [file mBio.02623-20-st004.docx]

| **Table S4. Analysis and visualization software** | | |
| --- | --- | --- |
| **SOFTWARE** | **SOURCE** | **VERSION** |
| Trimmomatic | (1) | version 0.32 |
| Cutadapt | (2) | version 1.5 |
| STAR | (3) | version 2.5.2a |
| ClipToolKit | (4) | <http://zhanglab.c2b2.columbia.edu/index.php/CTK> |
| Samtools | (5) | version 1.1 |
| Picard | http://broadinstitute.github.io/picard | version 1.141 |
| Bedtools | (6) | version 2.28.0 |
| R | (7) | version 3.5.2 |
| RStudio | (8) | version 1.1.463 |
| Gviz | (9) | version 1.26.4 |
| R4RNA | (10) | Version 1.10.0 |
| RNAfold | (11) | <http://rna.tbi.univie.ac.at/cgi-bin/RNAWebSuite/RNAfold.cgi> |
| VARNA | (12) | Version 3.9 |
| MetaXpress | Molecular Devices Corporation |  |

References:

1. Bolger AM, Lohse M, Usadel B. 2014. Trimmomatic: A flexible trimmer for Illumina sequence data. Bioinformatics 30:2114-2120.

2. Martin M. 2011. Cutadapt removes adapter sequences from high-throughput sequencing reads. EMBnetjournal 17:10-10.

3. Dobin A, Davis CA, Schlesinger F, Drenkow J, Zaleski C, Jha S, Batut P, Chaisson M, Gingeras TR. 2013. STAR: ultrafast universal RNA-seq aligner. Bioinformatics (Oxford, England) 29:15-21.

4. Shah A, Qian Y, Weyn-Vanhentenryck SM, Zhang C. 2017. CLIP Tool Kit (CTK): A flexible and robust pipeline to analyze CLIP sequencing data. Bioinformatics 33:566-567.

5. Li H, Handsaker B, Wysoker A, Fennell T, Ruan J, Homer N, Marth G, Abecasis G, Durbin R. 2009. The Sequence Alignment/Map format and SAMtools. Bioinformatics 25:2078-9.

6. Quinlan AR, Hall IM. 2010. BEDTools: a flexible suite of utilities for comparing genomic features. Bioinformatics (Oxford, England) 26:841-842.

7. Team RC. 2018. R: A Language and Environment for Statistical Computing, v20. R Foundation for Statistical Computing, Vienna, Austria. https://r-project.org/.

8. Team RS. 2016. RStudio: Integrated Development for R, RStudio, Inc., Boston, MA. http://www.rstudio.com/.

9. Hahne F, Ivanek R. 2016. Visualizing Genomic Data Using Gviz and Bioconductor. Methods Mol Biol 1418:335-51.

10. Lai D, Proctor JR, Zhu JY, Meyer IM. 2012. R-CHIE: a web server and R package for visualizing RNA secondary structures. Nucleic Acids Res 40:e95.

11. Gruber AR, Lorenz R, Bernhart SH, Neubock R, Hofacker IL. 2008. The Vienna RNA Websuite. Nucleic Acids Research 36:W70-W74.

12. Darty K, Denise A, Ponty Y. 2009. VARNA: Interactive drawing and editing of the RNA secondary structure. Bioinformatics 25:1974-5.
